# The Effect of Sleep Environment on Sleep Quality and Behavior in Firefighters: A Cross-Sectional Study

**DOI:** 10.3390/ijerph23060692

**Published:** 2026-05-23

**Authors:** Jacquelyn N. Zera, Erica Esper, Anna Peluso Simonson, Ashley N. Clausen, Serena Paterno

**Affiliations:** 1 Department of Exercise Science, John Carroll University, University Heights, OH 44118, USA; erica.esper@stonybrook.edu (E.E.); asimonson@jcu.edu (A.P.S.); paterno.10@osu.edu (S.P.); 2 Marion Bloch Neuroscience Institute, Saint Luke’s Healthcare System, Kansas City, MO 64111, USA; aclausen@saint-lukes.org

**Keywords:** firefighters, sleep quality, sleep hygiene, shift work, occupational health

## Abstract

**Highlights:**

**Public health relevance—How does this work relate to a public health issue?**

- Sleep deprivation in first responders is a significant occupational hazard linked to chronic systemic inflammation, cardiovascular disease, and metabolic dysfunction.
- Impaired cognitive function from poor sleep quality compromises both firefighter safety and public emergency response effectiveness.

**Public health significance—Why is this work of significance to public health?**

- This study identifies that operational “on-call” stressors significantly override the benefits of private sleeping quarters, as individual dorms showed no improvement over communal bunks.
- While home sleep is superior to on-duty sleep, even off-duty sleep quality in this population frequently fails to meet clinical “good sleep” thresholds.

**Public health implications—What are the key implications or messages for practitioners, policy makers and/or researchers in public health?**

- Departmental resources should prioritize station-wide noise reduction protocols and “safety nap” policies rather than investing solely in physical remodeling of sleeping quarters.
- Behavioral interventions, such as Cognitive Behavioral Therapy for Insomnia (CBT-I) tailored for first responders, are recommended to address the high rate of sleep medication use and chronic arousal.

**Abstract:**

Firefighters face high-stress occupational demands and irregular shift work that negatively impact sleep quality, which is intrinsically linked to long-term physical and psychological health. This cross-sectional study examines how the physical sleep environment (home vs. work) and station sleeping arrangements (bunk-style vs. individual dorm-style quarters) influence subjective sleep quality in this population. Sixty-six career firefighters (Age = 40.89 ± 11.05 years), completed the Pittsburgh Sleep Quality Index (PSQI) to assess their sleep in both home and fire station environments, with data analyzed using Wilcoxon Signed Ranks and Mann–Whitney U tests. The results reveal significant differences (*p* < 0.001), with sleep duration, efficiency, subjective quality, and global PSQI scores all performing significantly better at home than at work. Notably, no significant differences were found between bunk-style and dorm-style sleeping quarters at the station. These findings suggest that firefighters experience poorer sleep while on duty regardless of room design, indicating that operational stressors like call volume and nocturnal arousal may be more influential on sleep quality than the physical arrangement of sleeping quarters, and could inform organizational policies and wellness programs aimed at reducing occupational fatigue.

## 1. Introduction

Firefighting is an inherently demanding profession that places individuals under persistent physical, psychological, and emotional stress [1]. The occupational demands extend far beyond the physical exertion of emergency response activities, with firefighters routinely exposed to high-stakes situations that require rapid decision-making and mental acuity, often under extreme environmental conditions. These demands, when combined with the unpredictable nature of emergency calls, create a work environment that is both physically exhausting and psychologically taxing. Compounding these stressors is the irregular nature of firefighters’ work schedules. Firefighters commonly work 24 h shifts, characterized by frequent nighttime emergency responses that result in extended wakefulness and repeated sleep interruptions [2]. This shift structure not only disrupts the body’s natural circadian rhythms but also interferes with the essential stages of restorative sleep required for physiological recovery [3].

The resulting sleep dysregulation and poor sleep quality has been associated with increased risk for a range of adverse health and occupational outcomes [2,4,5]. Physiologically, the impact of sleep loss is particularly hazardous in this population due to the “alarm surge”, or the rapid transition from a resting state to maximal physical exertion upon receiving a call. This acute sympathetic surge, when layered over the chronic cardiovascular and metabolic strain caused by insufficient sleep, contributes to an elevated risk for cardiovascular disease, metabolic dysfunction, and compromised immune function [6,7,8,9,10,11]. Psychologically, inadequate sleep is associated with chronic stress and an increased prevalence of mood disturbances, including anxiety and depression [12]. Regarding occupational performance, the cognitive deficits resulting from sleep fragmentation, such as impaired attention, reduced short-term memory, and slowed reaction times, pose a direct threat to fireground safety [12]. In an environment where seconds matter, reduced vigilance and slower reflexes increase the statistical likelihood of errors, accidents, and on-the-job injuries [13,14,15,16]. For firefighters operating in these time-sensitive contexts, these deficits have serious consequences for both personal safety and the public they serve.

Although the negative health impacts of poor sleep in this population are well-documented, the factors contributing to sleep disruption remain complex and multifactorial. Some research has indicated that psychological (anxiety), situational (sedentary lifestyle), and occupational (role of paramedic) elements affect sleep in firefighters [17]. However, despite the growing awareness of sleep disorders within the fire service, the physical infrastructure of the fire station itself remains an under-examined variable. Anecdotal reports and qualitative data suggest that the fire station sleep environment is inherently disruptive [18]. Environmental noise, alert tones, lighting, temperature, and the communal nature of sleeping arrangements can all prevent the attainment of deep, restorative sleep.

Fire stations vary widely in their architectural design and sleeping accommodations. Traditional stations often utilize open, communal “bunk-style” quarters, while modern facility designs are increasingly shifting toward individual “dormitory-style” rooms to enhance privacy and reduce environmental disturbances. While it is reasonable to hypothesize that more private and quiet sleeping environments would lead to better sleep outcomes and improved recovery, few studies [18,19] have empirically compared these conditions or assessed the degree to which they differ from the baseline of a home sleep environment.

To address this gap, the present study aimed to evaluate how sleep location and fire station infrastructure influence subjective sleep quality and behavior among professional firefighters. Specifically, the objectives of this study were to compare subjective sleep quality, duration, and efficiency between (1) the sleep location (home vs. work), and (2) on duty sleep environment (bunk-style vs. dorm-style). This investigation serves as a necessary step in understanding the complex factors that influence sleep in a high-stress occupational setting. The results will provide actionable insights into how fire station infrastructure contributes to sleep health, helping to inform organizational policies and wellness programs aimed at reducing occupational fatigue.

## 2. Materials and Methods

### 2.1. Participants

A convenience sample of sixty-six career firefighters (Age = 40.89 ± 11.05 years), employed across two departments and three stations, participated in this study. Participants were drawn from a larger cohort enrolled in an ongoing, department-wide employee wellness community project. All departments included in the current investigation utilize a 3-day shift cycle, where firefighters complete 24 h on duty, followed by 48 h off duty. Inclusion criteria included current employment as a firefighter and/or administrator in the department and completion of the department’s annual health and fitness evaluation. All participants provided informed consent for their data to be included in a secure research repository for future analysis. Institutional review board exemption for the retrospective use of this secondary data was obtained on 12 July 2022 (IRB 2023-004).

### 2.2. Procedure

This study utilized a cross-sectional, retrospective design analyzing secondary data obtained from the community wellness project repository. As part of their mandatory annual health and fitness evaluations requested by the city and respective departments in the Spring of 2023, participants completed a health history questionnaire and the Pittsburgh Sleep Quality Index (PSQI) in a digital format via a Qualtrics survey (Qualtrics, Provo, UT). The assessment was conducted in a single session at the beginning of each participant’s evaluation. To isolate the effects of the environment, the PSQI was administered twice within the same survey: once with instructions to reflect on typical sleep at home, and once for sleep while on duty at the fire station.

### 2.3. Measures

Anthropometrics: As part of the annual health and fitness evaluation, physical characteristics and clinical vitals were collected by certified exercise physiologists.

Height and Weight: Body mass index (BMI) (kg/m^2^) was calculated using measured height and weight. Height was measured using a wall-mounted stadiometer to the closest quarter inch (Seca, Hamburg, Germany). Weight was measured with participants wearing lightweight exercise clothing and shoes removed, using a Model 349KLX Health-o-Meter Professional digital scale (Pelstar, LLC., Health o meter Professional, McCook, IL, USA), revisedrecorded to the nearest tenth of a pound.

Blood Pressure: Blood pressure (mmHg) was measured after the subject had rested for a minimum of 5 min in a chair with back support and feet flat on the floor. Measurements were taken via auscultation using an appropriately sized blood pressure cuff, a sphygmomanometer, and a Littmann Classic III stethoscope (3M, Columbia, MO, USA) placed over the brachial artery of the right arm. One measurement was recorded by one of two certified exercise physiologists.

Body Composition: Percent body fat (%) was assessed using bioelectrical impedance analysis to the nearest tenth of a percentage using a Model HBF-306C Omron handheld monitor (OMRON Global, Kyoto, Japan).

Sleep Quality: The PSQI is a widely used, validated self-report measure of subjective sleep quality over a one-month interval. It consists of seven components: subjective sleep quality, sleep latency, sleep duration, habitual sleep efficiency, sleep disturbances, use of sleep medication, and daytime dysfunction. Component scores are summed to produce a global PSQI score on a scale or 0–21, with higher scores indicating poorer sleep quality. A PSQI global score ≤ 5 points is classified as a good sleep quality group, and PSQI global score > 5 is classified as a poor sleep quality group [20].

Sleep Environment: Environment type was reported by each firefighter (administrators not included). At two of the fire stations, firefighters reported bunk-style sleeping quarters (n = 35), and one reported dorm-style quarters (i.e., individual rooms; n = 24).

### 2.4. Statistical Analysis

All data were analyzed using SPSS 30.0 (IBM Corp., Armonk, NY, USA). Normality of the Global PSQI score and PSQI sub-scores were assessed using the Shapiro–Wilk test. Due to non-normal distribution of data, nonparametric tests were employed. A Wilcoxon Signed Ranks Test was used to compare within-subject differences in the Global PSQI score and PSQI sub-scores between home and work sleep environments. A Mann–Whitney U Test was used to assess between-group differences in the Global PSQI score and PSQI sub-scores based on sleeping quarter type (bunk vs. dorm). Statistical significance was set at *p* < 0.05 a priori.

## 3. Results

### 3.1. General Physical Health

Subjects of the current investigation (*N* = 66) are characterized by a high prevalence of overweight or obesity, with a mean BMI of 29.01 kg/m^2^ (*SD* = 3.84), and elevated blood pressure with a mean SBP of 128.06 mmHg (*SD* = 9.63) and DBP of 76.70 mmHg (*SD* = 7.40 (Table 1). This is consistent with the general population in which 72.5% of adults over the age of 20 categorized as overweight [21] with an average blood pressure of 123/73 mmHg [22].

### 3.2. Overall Sleep Quality

Analysis of PSQI scores indicated that overall, firefighters experience poor sleep quality regardless of sleep environment. Mean Global PSQI scores were 7.34 ± 3.30 for sleep at work and 5.66 ± 3.24 for sleep at home.

### 3.3. Associations Between Sleep Location and Sleep Quality

**Figure 1:**
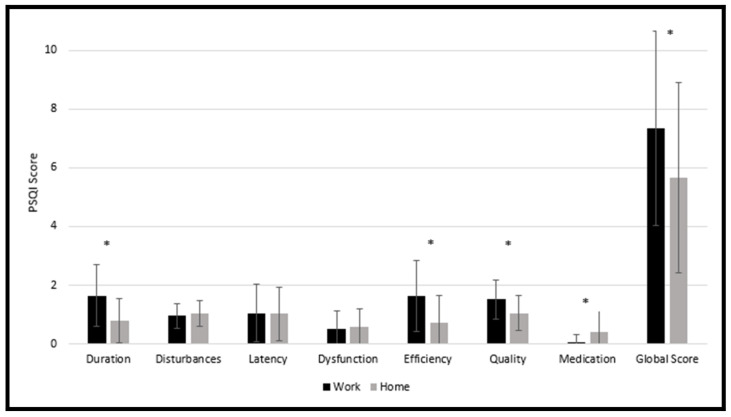
Rank-order differences in PSQI components and Global scores between sleep locations. * Indicates statistical significance at *α* < 0.05.

Results of multiple Wilcoxon Signed Ranks Tests demonstrate significant differences in multiple sleep parameters between home (off-shift) and work (on-shift) locations. Firefighters reported significantly better sleep duration (Z = −5.078, *p* < 0.001), sleep efficiency (Z = −3.991, *p* < 0.001), subjective sleep quality (Z = −4.466, *p* < 0.001), and Global PSQI Score (Z = −4.424, *p* < 0.001) sleeping at home compared to sleeping at work. There were no reported significant differences between sleep at home and at work for sleep latency, sleep disturbances, or daytime dysfunction (all *p* > 0.05). Finally, a significant difference in use of sleep medication was identified (Z = −3.111, *p* = 0.002), although it should be noted that firefighters are prohibited from using prescription sleep medication on-shift (see Table 2 and Figure 1).

### 3.4. Associations Between Sleep Environment and Sleep Quality

Results of Mann–Whitney U tests indicate no significant differences in any PSQI components or Global PSQI scores between firefighters sleeping in bunk-style vs. dorm-style quarters at work (all *p* > 0.05).

## 4. Discussion

The primary objective of this study was to evaluate the influence of the physical sleep environment, specifically comparing home vs. on-duty locations and bunk-style vs. dorm-style station quarters, on the subjective sleep quality of professional firefighters. By examining these variables, we sought to determine if more modern station infrastructure to provide more private accommodations (dorm-style) significantly mitigates the sleep dysregulation common in this high-risk occupation, and to inform future organizational policies and wellness programs aimed at reducing occupational fatigue.

Consistent with prior research [2,19] the current findings demonstrate that firefighters experience significantly shorter sleep duration and poorer sleep efficiency while on duty compare to when at home. While sleep at home was statistically superior, it is noteworthy that global PSQI scores in both environments remained above the clinical threshold for “poor” sleep. This suggests that the physiological and psychological stressors of firefighting create a baseline of sleep debt that is not fully recovered during off-duty periods, and is consistent with previous research [3]. Critically, poor sleep, including insomnia, has been shown to increase the risk of cerebrovascular and cardiometabolic disease, such as myocardial infarction and stroke, in firefighters [23,24]. Thus, interventions targeting sleep quality at home and on shift will be vital to improving firefighters’ health. Interestingly, the current investigation identified a higher rate of sleep medication use reported at home, which may be a compensatory behavior for the chronic sleep fragmentation experienced on shift. As suggested by recent literature, the “alarm surge” and unpredictable call volumes during 24 h shifts create a state of hyper-arousal [10,24]. Our results imply that this distress “spills over” into the home environment, where firefighters may rely on pharmacological aids to force a state of rest that their natural circadian rhythms, disrupted by shift work, can no longer easily achieve.

Another key objective of this study was to assess whether individual dormitory-style rooms improved sleep outcomes compared to traditional open bunk-style quarters. Contrary to our hypothesis, no significant differences were identified between these two station environments. This finding suggests that the operational stressors, such as emergency tones, high call volumes, and the “first-night effect” of sleeping in a state of constant readiness, exert a much stronger influence on sleep quality than the physical design of the room [18]. While modernizing stations to provide private dorms offers benefits for privacy and morale, our data indicate that room design alone is insufficient to counteract the sleep fragmentation inherent in a 24/48 shift schedule. This aligns with recent studies suggesting that recovery between shifts is often truncated in 24 h rotations, regardless of the physical comfort provided [25].

A subset of literature investigating sleep in firefighters suggests the nature of shift work can have a significant influence on sleep patterns and overall sleep health. When examining the length of shift, 48 h on shift, followed by 96 h off was associated with the best sleep and health outcomes, allowing firefighters to establish more routine sleep habits and allowed for recovery between shifts [25]. However, as in this current population, fire departments commonly operate on a 24 h on and 48 h off shift schedule (24/48), potentially impacting recovery. It is possible that the chronic sleep fragmentation experienced during these shifts leads to persistent psychological distress that extends into off-duty days, potentially necessitating the use of pharmacological sleep aids or medications at home to manage impaired sleep quality. This suggests that poor occupational sleep may create a cycle of dysregulation that is reflected in the home environment even when the immediate environmental stressors of the station are removed. In response, safety naps, or intra-shift napping, can help reduce fatigue associated with first responder shift work, improve recovery between shifts, improve mood, and regulate blood pressure [26].

Educational programs have also shown promise at improving overall sleep quality. These programs typically include education on sleep hygiene, occupational factors that can negatively affect sleep, as well as ways to address co-morbid or contributing factors to sleep quality such as mental health symptoms, BMI, or chronic pain. While there is strong support for implementation of educational programs aimed at improving sleep quality, further studies investigating specific interventions have been recommended [27]. As such, the null difference in sleep quality by sleep environment suggests that interventions to improve sleep among firefighters should focus more broadly on station-wide sleep protocols, noise reduction, and recovery strategies rather than simply modifying room design. Additionally, treatments such as Cognitive Behavioral Therapy for Insomnia, or Imagery Rehearsal Therapy, which target insomnia and nightmares in firefighters may be an effective way to improve sleep quality and quantity in on-shift firefighters [28].

This study is limited by its cross-sectional design and reliance on self-reported sleep data, which may lead to recall bias or a lack of precision in distinguishing home vs. work sleep quality. Objective measures such as actigraphy or polysomnography would be beneficial to inform and tailor future interventions in this population. Additionally, factors such as call volume, individual station culture, noise levels, and psychosocial stress were not controlled, and may confound the observed relationships. Importantly, firefighters were asked to evaluate sleep over the last 30 days, which was split between sleep at home and on shift. While the PSQI evaluates sleep over the last 30 days, participants were not evaluating 30 days of sleep at home versus 30 days of sleep on shift due to the nature of their schedules. As such, it is possible that PSQI scores may not accurately reflect overall sleep quality in the past month. Future research should utilize longitudinal designs and objective measures, such as actigraphy or polysomnography, to capture real-time physiological recovery. Investigating the impact of automated “quiet” dispatch systems (which alert only the necessary crew) rather than station-wide tones may also provide more insight into noise-related disruption than room architecture.

## 5. Conclusions

This study concludes that while firefighters sleep better at home than at work, the physical transition from bunk-style to dorm-style quarters does not significantly improve subjective sleep quality. The over-riding impact of operational demands and shift-work-related distress appears to nullify the potential benefits of improved architectural privacy.

The practical implications of these findings are twofold. First, fire department leadership should recognize that infrastructure upgrades alone are not a “silver bullet” for the sleep crisis in the fire service. Rather than focusing solely on station remodeling, resources should be directed toward comprehensive sleep health protocols, including the implementation of intra-shift “safety naps,” noise-reduction technologies, and access to specialized behavioral therapies (e.g., CBT-I). Second, because poor sleep health persists even in the home environment, wellness programs must emphasize off-duty recovery strategies and the risks of long-term reliance on sleep medications. Prioritizing these evidence-based interventions is essential for mitigating the cardiovascular and metabolic risks that threaten the longevity of the firefighting workforce.

## Figures and Tables

**Table 1:** Demographic characteristics.

Characteristic	*M* ± *SD*
Age (years)	4089 ± 11.05
Body Mass Index (kg/m^2^)	29.01 ± 3.84
Resting Blood Pressure (mmHg)	
SBP	128.06 ± 9.63
DBP	76.70 ± 7.40
Percent Body Fat (%)	22.61 ± 6.35
Waist-to-Hip Ratio	0.92 ± 0.07

**Table 2:** Mean differences in PSQI component scores between sleep locations (* *p*-value < 0.001).

PSQI Component	Work *M* ± *SD*	Home *M* ± *SD*	*p*-Value	Effect Size (*r*)
Sleep Duration	1.64 ± 1.05	0.80 ± 0.75	<0.001 **	0.42
Sleep Disturbances	0.95 ± 0.43	1.03 ± 0.43	0.10	0.39
Sleep Latency	1.05 ± 0.99	1.02 ± 0.92	0.28	0.27
Daytime Dysfunction	0.52 ± 0.60	0.58 ± 0.63	0.00	0.20
Sleep Efficiency	1.62 ± 1.21	0.72 ± 0.93	<0.001 **	0.32
Sleep Quality	1.51 ± 0.67	1.05 ± 0.59	<0.001 **	0.41
Sleep Medication	0.05 ± 0.28	0.39 ± 0.84	0.002 *	0.27
PSQI Total	7.34 ± 3.30	5.66 ± 3.24	<0.001 **	0.44

* Indicates statistical significance at *α* < 0.05; ** indicates a statistical significance of *α* < 0.001.

## Data Availability

The datasets presented in this article are not readily available because the data is part of an existing database of an ongoing study. Requests to access the datasets should be directed to Jacquelyn Zera.

## Abbreviations

The following abbreviations are used in this manuscript:

PSQI	Pittsburgh Sleep Quality Index
